# Melittin alleviates bleomycin-induced pulmonary fibrosis *in vivo* through regulating TGF-β1/Smad2/3 and AMPK/SIRT1/PGC-1α signaling pathways 

**DOI:** 10.22038/ijbms.2024.81986.17740

**Published:** 2025

**Authors:** Jia-Wang Yu, Wei-Hua Lu

**Affiliations:** 1 The Fifth Clinical Medical College of Anhui Medical University, Hefei, 230032, China; 2 EICU, The First Affiliated Hospital of Wannan Medical College (Yijishan Hospital of Wannan Medical College), Wuhu, 241000, China; 3 ICU, The First Affiliated Hospital of Wannan Medical College (Yijishan Hospital of Wannan Medical College), Wuhu, 241000, China

**Keywords:** Epithelial-mesenchymal transition, Melittin, Mitochondria, Oxidative stress, Pulmonary fibrosis

## Abstract

**Objective(s):**

The present study investigated the protective effect of melittin (MEL) against bleomycin (BLM)- induced pulmonary fibrosis (PF) in mice and the mechanism underlying this effect.

**Materials and Methods:**

A mouse model of PF was established by intratracheal injection of 3.5 mg/kg BLM. Twenty-four hours after the model was established, the mice in the treatment groups were intraperitoneally injected with MEL, and specimens were collected 28 days later. The body weight, survival rate, and pulmonary index (PI) of the mice were determined. Haematoxylin and eosin (HE) staining, Masson’s trichrome staining, immunohistochemical staining, kit assays, and Western blot (WB) analysis were performed.

**Results:**

Our study indicated that MEL significantly increased the body weight and survival rate, reduced PI, and improved lung histopathology in mice. In addition, MEL inhibited epithelial-mesenchymal transition (EMT) and extracellular matrix (ECM) deposition. Attenuated mitochondrial damage and reduced oxidative stress (OS) were also observed in MEL-treated mice. We further showed that MEL inhibited the TGF-β1/Smad2/3 pathway and activated the AMPK/SIRT1/PGC-1α pathway.

**Conclusion:**

MEL is a promising future therapeutic agent for PF. Its multifaceted and complex mechanism of action inhibits both EMT and ECM production by modulating the TGF-β1/Smad2/3 pathway. It also improves mitochondrial function and reduces OS at least partially through the activation of the AMPK/SIRT1/PGC-1α signaling pathway.

## Introduction

Pulmonary fibrosis (PF) is an end-stage change in various lung diseases. It is characterized by progressive difficulty breathing, lung ventilation dysfunction, inflammatory injury, and structural destruction of the lung parenchyma (1). The clinical progression of PF is variable and difficult to predict, and the pathogenesis of this disease has not been determined. Although pirfenidone and nintedanib are currently available clinical agents for treating PF, neither has been shown to improve survival or quality of life (2). Hence, searching for effective therapeutic agents and potential action targets for PF is crucial.

The critical roles of abnormal epithelial cell-mediated damage repair, epithelial-mesenchymal transition (EMT), fibroblast/myofibroblast activation, and extracellular matrix (ECM) accumulation in the initiation of PF are widely recognized (3). Multiple signaling pathways, including Smad-dependent and Smad-independent, are involved in TGF-β1-induced EMT (4). Accumulating evidence suggests that TGF-β1 activity depends predominantly on the canonical Smad signaling pathway. TGF-β1 induces the phosphorylation of Smad2/3, which subsequently forms a trimer with Smad4; the trimer then translocates to the nucleus to regulate target gene expression.

It is universally accepted that the degree of oxidative stress (OS) is closely correlated with PF severity (5). As the main site of energy production through the oxidative respiratory chain, mitochondria are also the main source of reactive oxygen species (ROS) (6). Metabolic reprogramming, with decreases in mitochondrial oxygen consumption, respiration, and electron transport chain (ETC) activity, occurs in models of bleomycin (BLM)-induced PF (7,8). The AMPK/SIRT1/PGC-1α pathway is an energy-sensing network that plays important roles in mitochondrial biosynthesis, energy metabolism, and the OS defense (9). AMPK is a serine/threonine kinase that has played an important role in maintaining intracellular energy homeostasis throughout eukaryotic evolution, and it is activated in response to altered energy ratios caused by defects in energy production or increased energy expenditure (10). Once activated, AMPK activates catabolic pathways to produce adenosine triphosphate (ATP) while terminating energy-consuming anabolic processes. During energy deficit, AMPK increases the expression of genes related to glucose transport, glycolysis, and mitochondrial respiration while downregulating lipid synthesis genes. Activation of AMPK, in turn, activates SIRT1 by increasing the intracellular nicotinamide adenine dinucleotide (NAD^+^) level (11). Activated AMPK and SIRT1 directly or indirectly affect the activity of PGC-1α through phosphorylation and deacetylation, respectively (12).

Melittin (MEL), also called bee venom (BV) hemolytic peptide, is a small-molecule peptide biotoxin that accounts for approximately 50% of the dry weight of BV. MEL is the main functional substance in BV and has antibacterial, antiviral, anti-inflammatory, antitumor, and antirheumatic effects (13,14). EI-Aarag *et al*. (15) state that MEL inhibits OS, exerts antiapoptotic effects, and may be an effective drug for treating paraquat-induced lung injury. A study (16) showed that MEL reduced the levels of phosphorylated Smad2/3 directly or indirectly by reducing the expression level of TRIM47, thus exerting an antifibrotic effect on human embryonic lung fibroblasts (HELFs). Park *et al*. (17) found that MEL exerts potent antifibrotic and anti-EMT effects via inhibition of the TGFβ/Smad and TGFβ/Smad-independent c-Jun N-terminal kinase (JNK)/Mitogen-activated protein kinase (MAPK) signaling pathways.

In this study, we examined whether MEL inhibits EMT and the production of ECM by modulating the TGF-β1/Smad2/3 pathway, improves mitochondrial function, and reduces OS through the activation of the AMPK/SIRT1/PGC-1α signaling pathway, thereby ameliorating BLM-induced PF in mice. Key target genes were identified, providing an important experimental basis for screening suitable drugs for PF treatment.

## Materials and Methods

### Experimental animals and reagents

Specific pathogen-free (SPF)-grade male C57BL/6J mice weighing 22 ± 2 g were purchased from Tianqin Biotechnology Co., Ltd (Changsha, China; experimental animal certificate number: SCXK (Xiang) 2019-0014). MEL was purchased from Shanghai Aladdin Biochemical Technology Co., Ltd (Shanghai, China). BLM was obtained from Hefei Bomei Biotechnology Co., Ltd (Hefei, China). Mitochondrial respiratory chain complex I-V and ATP detection kits were acquired from Beijing Boxbio Science & Technology Co., Ltd (Beijing, China). Total anti-oxidant capacity (T-AOC), glutathione peroxidase (GSH-PX), malondialdehyde (MDA), and hydroxyproline (HYP) detection kits were obtained from Nanjing Jiancheng Bioengineering Institute (Nanjing, China). Anti-α-SMA and anti-TGF-β1 antibodies were procured from Bioworld Technology Co., Ltd (Nanjing, China). Anti-Collagen I, anti-Collagen III, anti-Ecadherin, anti-Vimentin, anti-Smad2/3, and anti-p-Smad2/3 antibodies were purchased from Affinity Biosciences (Changzhou, China). Antibodies specific for AMPK, p-AMPK, SIRT1, and PGC-1α were purchased from Abcam (UK). The anti-GAPDH antibody was purchased from Beyotime Biotechnology Co., Ltd (Shanghai, China).

### Animal model establishment and grouping

After adaptive feeding for one week, male C57BL/6J mice were randomly divided into five groups: the control group, the PF model group, and the low-dose (0.05 mg/kg), medium-dose (0.1 mg/kg), and high-dose (0.2 mg/kg) MEL groups. The mice were given a single intratracheal injection of BLM (3.5 mg/kg) to establish the PF model. The indicated doses of MEL were intraperitoneally injected twice weekly for 28 days, starting on the second day after modeling. Specimens were collected for further analysis. All animals were housed under SPF conditions (24±2 °C, 60 ± 10% relative humidity, and alternating 12 hr dark/light cycles). Standard food and water were provided *ad libitum* during the experiment. The experimental procedures adhered to the principles outlined in the Experimental Animal Care and Use Guide. All procedures were approved by the Committee on the Ethics of Animal Experiments of Wannan Medical College (No: LLSC-2022-253, March 23, 2023).

### Body weight and survival rate measurements, evaluation of lung morphology, and determination of the pulmonary index (PI)

Body weight was recorded from the first day of modeling and every three days thereafter until day 28. Twenty-four hours after the final drug administration, the mice were sacrificed. The lungs were quickly harvested, rinsed with precooled normal saline solution, and dried with filter paper. They were then photographed and weighed. The PI was calculated as the whole-lung weight/body weight × 100%. 

### Histopathological evaluation

The left lungs were uniformly harvested and fixed with 4% paraformaldehyde for routine hematoxylin and eosin (HE) staining and Masson’s trichrome staining. The degree of alveolitis and fibrosis in each sample was evaluated by light microscopy and scored according to the methods described by Szapiel *et al*. (18).

### Kit assays

HYP concentrations were measured according to the protocol of the HYP assay kit. The absorbance of each sample was measured at 550 nm and used to calculate the HYP concentration. The serum T-AOC was determined by the iron reduction method, serum GSH-PX activity was determined by the dithio-bis-nitrobenzoic acid method, and serum MDA was quantified by the thiobarbituric acid method. After sample preparation, measurements were conducted using a multifunctional microplate reader. Mitochondrial respiratory chain complex I-V activity and ATP levels were measured using the appropriate test kits.

### Immunohistochemical analysis

Mouse lung tissue sections were routinely deparaffinized and dehydrated through an ethanol gradient. Antigen retrieval was performed in citrate buffer, and endogenous peroxidase activity was quenched with 3% hydrogen peroxide. The sections were incubated with primary antibodies specific for α-SMA (1:100) and TGF-β1 (1:100) at 4 °C overnight and were then washed with PBS and incubated with secondary antibodies (1:100) at 37 °C for 60 min. Then, the sections were washed with PBS, stained with 3,3’-diaminobenzidine (DAB), counterstained with hematoxylin, and mounted. Positive protein expression was detected via optical microscopy, and staining was analyzed using Image J software. Six different fields of view were randomly selected for each group of lung tissue sections, and the average optical density (AOD) was measured and recorded to determine the degree of positive expression.

### Western blot (WB) analysis

The concentration of extracted proteins was determined by the bicinchoninic acid (BCA) method, and denatured proteins in the samples were separated by sodium dodecyl sulfate-polyacrylamide gel electrophoresis (SDS–PAGE). After electrophoresis, the proteins were transferred to a polyvinylidene fluoride (PVDF) membrane. The PVDF membrane was blocked in 5% skim milk or 5% bovine serum albumin (BSA) at four °C for 2 hr. It was then incubated with diluted primary antibodies (anti-α-SMA, anti-TGF-β1, anti-Collagen I, anti-Collagen III, anti-Ecadherin, anti-Vimentin, anti-Smad2/3, anti-p-Smad2/3, anti-AMPK, anti-p-AMPK, anti-SIRT1, anti-PGC-1α or anti-GAPDH) at 4 °C overnight. The membrane was then incubated with the corresponding secondary antibody at 4 °C for 2 hr. Eventually, the signals were detected using an enhanced chemiluminescence (ECL) system, and band densities were quantified using Image J software.

### Statistical analysis

The data are expressed as the mean ± standard deviation (SD; ±s). Differences among more than two groups were compared using one-way analysis of variance (ANOVA) with GraphPad Prism 8.0 software. Comparisons between the two groups were performed using the t-test. A *P*-value<0.05 indicated that a difference was statistically significant. Each experiment was repeated three times.

## Results

### MEL attenuated BLM-induced PF

**Figure 1:**
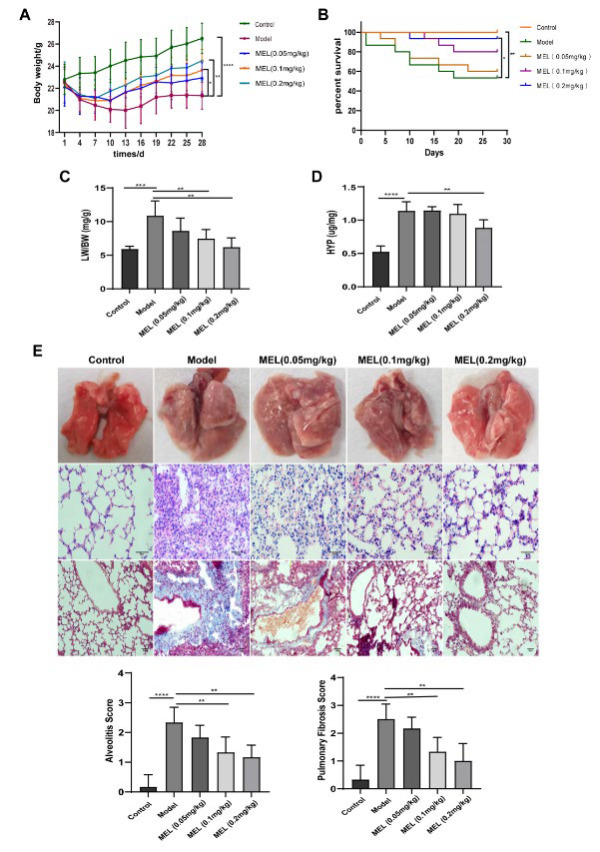
MEL attenuated BLM-induced PF in mice

As shown in Figure 1A-D, BLM-treated mice exhibited significantly reduced body weight and survival rate and significantly increased PI and HYP concentrations. However, these detrimental changes were ameliorated to varying degrees after MEL treatment, with 0.2 mg/kg MEL exerting the best effect. Representative lung images and images of HE and Masson’s trichrome staining showed that MEL treatment gradually resolved severe lung tissue lesions caused by BLM, as indicated by the improvements in lung morphology and alveolar structure, the decrease in inflammatory cell infiltration, and the reduction in collagen fiber deposition (Figure 1E).

### MEL inhibited ECM deposition and EMT in BLM-challenged mice

**Figure 2:**
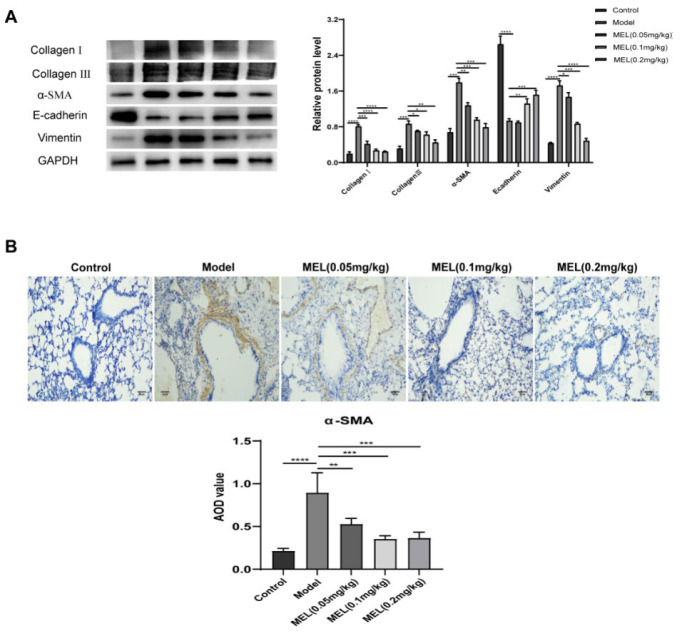
MEL inhibited ECM deposition and EMT in BLM-challenged mice

Treatment with MEL at three tested doses inhibited the production of major ECM markers (Collagen I and Collagen III) in a dose-dependent manner (Figure 2A). As expected, the protein expression levels of EMT biomarkers (α-SMA, E-cadherin, and Vimentin) in the lung tissue of mice with BLM-induced PF were also dramatically reversed by MEL treatment (0.2 mg/kg; Figure 2A). The immunohistochemical staining results for α-SMA were consistent with those of WB analysis (Figure 2B).

### MEL ameliorated BLM-induced OS and mitochondrial dysfunction

**Figure 3:**
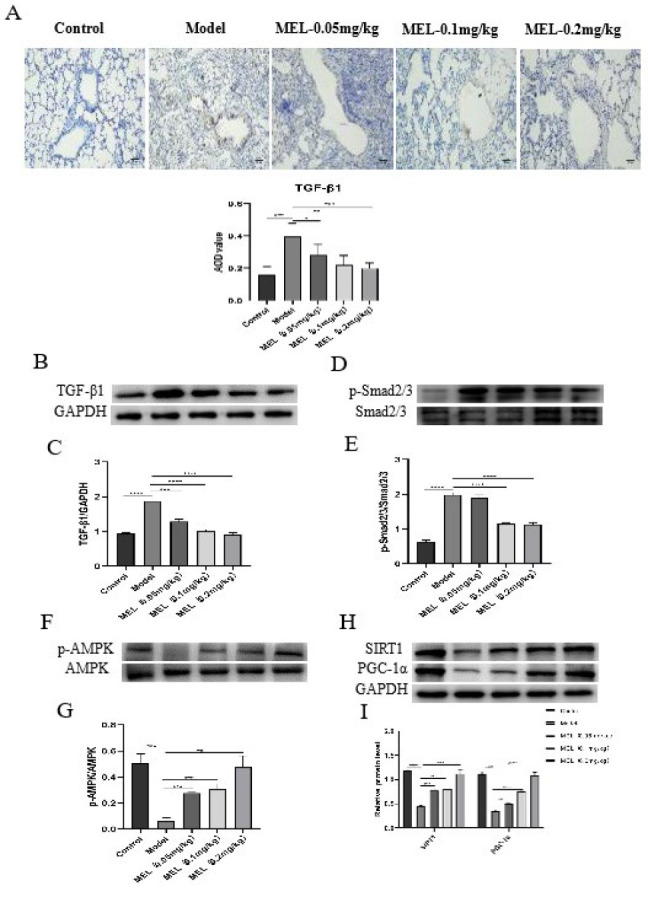
MEL ameliorated BLM-induced OS and mitochondrial dysfunction

We measured the serum T-AOC, GSH-PX activity, and MDA content to examine whether MEL ameliorates OS. The serum T-AOC and GSH-PX activity were significantly reduced (Figure 3A, B), but the MDA content (Figure 3C) significantly increased in the model group compared with the control group. However, after treatment with MEL (0.2 mg/kg), the changes in the above indicators were significantly ameliorated, and the values approached normal levels. 

In addition, we found that the important indicators of mitochondrial function, including mitochondrial respiratory chain complex I-V activity (Figure 3D-H) and the ATP level (Figure 3I) in the lung tissue, were markedly reduced in the model group, and these reductions were alleviated by MEL (0.2 mg/kg) treatment. 

### MEL exerted anti-PF effects by regulating the TGF-β1/Smad2/3 and AMPK/SIRT1/PGC-1α signalling pathways

**Figure 4:**
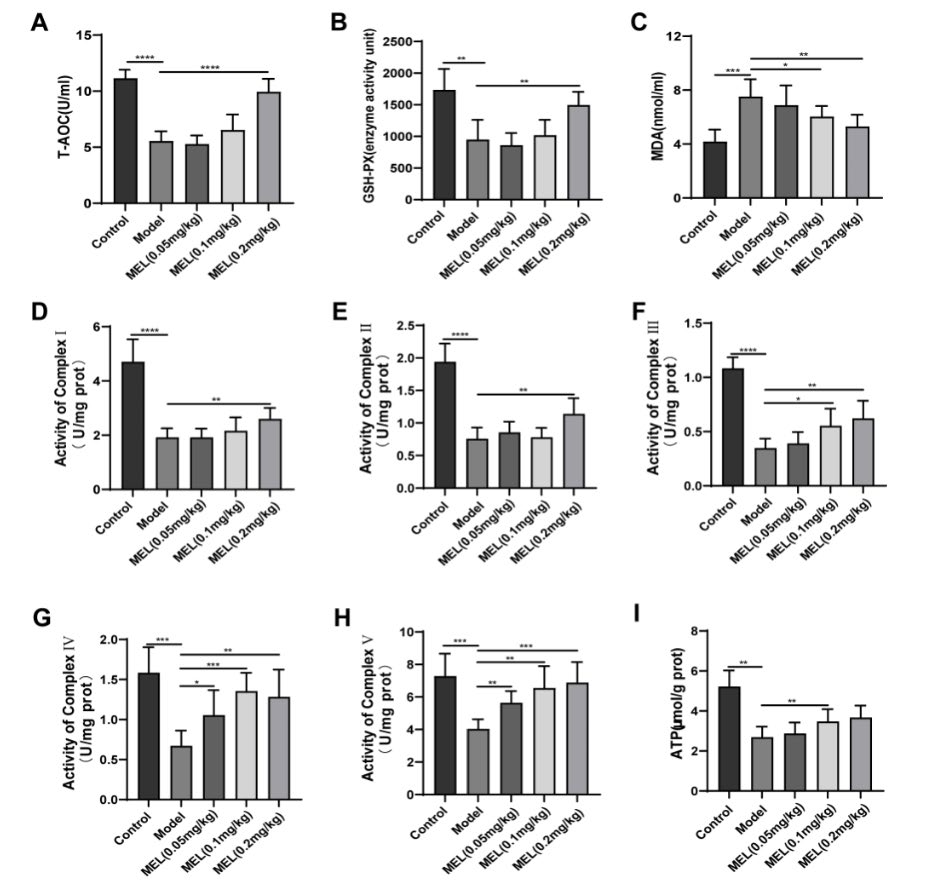
MEL exerted anti-PF effects by regulating the TGF-β1/Smad2/3 and AMPK/SIRT1/PGC-1α signaling pathways

To further investigate the potential mechanisms by which MEL exerts antifibrotic effects, the expression of proteins related to the TGF-β1/Smad2/3 and AMPK/SIRT1/PGC-1α signaling pathways was detected. WB analysis revealed a markedly higher level of TGF-β1, a markedly higher p-Smad2/3:Smad2/3 ratio (Figure 4B-E), but lower levels of p-AMPK, SIRT1, and PGC-1α and a lower p-AMPK:AMPK ratio (Figure 4F-I) in lung tissues of the model group, and these changes were ameliorated by MEL treatment. We also evaluated TGF-β1 expression by immunohistochemistry and found that it was significantly reduced in a dose-dependent manner after MEL treatment (Figure 4A).

## Discussion

PF is involved in a large group of lung diseases caused by diverse factors that induce alveolar epithelial cell (AEC) injury, apoptosis, and the activation of fibroblasts to myofibroblasts in distal lung interstitial tissue, and it is associated with copious ECM and collagen deposition (19). In this study, mice in the model group exhibited behavioral and physical changes, such as sluggishness, listlessness, reduced food intake, and hair loss; additionally, compared with those of control mice, their body weight and survival rate were significantly lower, while their PI was significantly higher. Moreover, histopathological analyses and measurement of the HYP concentration in tissues revealed obvious alveolar structural damage, inflammation, fibrosis, and collagen deposition, consistent with the results of previous studies (20, 21). These findings indicate that we successfully established a mouse model of PF. Notably, these pathological changes were ameliorated to varying degrees after treatment with different doses of MEL, with the most marked amelioration observed in the high-dose group, suggesting that MEL treatment can ameliorate BLM-induced PF. 

EMT, ECM production, and collagen deposition are three major sequential events that are closely associated with the development of PF (22). EMT is a complex process in which epithelial cells acquire a mesenchymal morphology via increased expression of mesenchymal markers and decreased expression of epithelial markers, accompanied by the deposition of large amounts of ECM. Collagen, glycoproteins, and polysaccharides are the main components of the ECM (23, 24). TGF-β1 is currently considered the primary cytokine that promotes PF (25). The binding of TGF-β1 to the TGF-β receptor results in the phosphorylation of Smad2/3, which subsequently promotes EMT and ECM accumulation (26). This led us to focus on the effect of MEL on the TGF-β1/Smad2/3 signaling pathway. Considerable evidence indicates that BLM leads to EMT, ECM deposition, and increased TGF-β1 expression, as well as Smad2/3 phosphorylation (27, 28). Our results were consistent with these findings. MEL has been reported to have potent antifibrotic effects in terms of both renal fibrosis and hepatic fibrosis and anti-EMT effects that are mediated by the TGF-β1/Smad2/3 pathway (17, 29). As expected, our results indicated that MEL significantly inhibits BLM-induced EMT and ECM deposition by modulating the TGF-β1/Smad2/3 signaling pathway.

Studies have shown that redox imbalance is closely related to the pathogenesis of PF and plays an important role in the pathological changes occurring during its development (30). Previous studies have demonstrated elevated lipid peroxidation and anti-oxidant deficiencies in an animal model of BLM-induced PF (31, 32). Studies have shown that MEL has an anti-OS effect on ulcerative colitis (33), neurodegenerative diseases (34), and acute lung injury (15). In our study, we confirmed the OS response, stimulated by BLM challenge, in lung tissue, as evidenced by decreased T-AOC and GSH-PX activity and increased MDA content; notably, these effects were reversed by MEL treatment.

Mitochondria, the key organelles for oxidative reactions in eukaryotes and known as the powerhouses of the cell, are essential for energy metabolism and aerobic respiration and are important sites for the generation of approximately 90% of intracellular ATP. Most of the energy needed for biological activities is produced in mitochondria through the ETC, also called the respiratory chain (35). The mitochondrial respiratory chain is composed of five enzyme complexes (NADH dehydrogenase, succinate dehydrogenase, the CoQH2-cytochrome C reductase complex, cytochrome C oxidase, and ATP synthase), which cooperate to accomplish cellular respiration. During the electron transfer process in the mitochondrial respiratory chain, the normal function of mitochondria depends on the integrity and activity of the enzymes in this chain (36, 37). Abnormal transmission of the mitochondrial respiratory chain has been found in PF and leads to mitochondrial damage (38). However, whether MEL can ameliorate the mitochondrial dysfunction caused by BLM in PF is unknown. Here, we found that MEL treatment increased the activity of mitochondrial respiratory chain complexes I-V and the level of ATP, maintaining mitochondrial bioenergetic function and thereby alleviating BLM-induced lung tissue lesions. AMPK is an energy sensor that is responsible for the maintenance of cellular energy homeostasis and plays a critical role in the defense against OS (39, 40). AMPK can directly phosphorylate PGC-1α but can also activate SIRT1 by increasing the NAD^+^ level, ultimately leading to PGC-1α deacetylation, thus ensuring an adequate energy supply, reducing OS and contributing to cellular recovery (41). The protective role of the AMPK-related axis in the lung has been a popular research topic (42). It has been shown that the AMPK/SIRT1 pathway plays an important role in BLM-induced PF (43, 44). However, no studies have confirmed whether MEL can act on the AMPK/SIRT1/PGC-1α signaling pathway. The present study suggested that MEL can activate the AMPK/SIRT1/PGC-1α signaling pathway by increasing the p-AMPK:AMPK ratio and the expression of SIRT1 and PGC-1α. 

Changes in body weight (A), survival rate (%) (B), and LW/BW ratio (C) are shown across the groups. (D) HYP content in lung tissues from the different groups. (E) Representative images from the macroscopic lung examination and HE- and Masson's trichrome-stained lung sections. Experienced pathologists scored inflammation and fibrosis in a blinded manner. The data are the mean ± SD (n =6). *P<0.05, **P<0.01, ***P<0.001, ****P<0.0001.

MEL: Melittin; BLM: Bleomycin; PF: Pulmonary fibrosis; HYP: Hydroxyproline; HE: Haematoxylin and eosin

Expression levels of Collagen I, Collagen III, α-SMA, E-cadherin, and Vimentin in lung tissues were examined by WB and quantitative analyses (A). Immunohistochemistry was performed to assess α-SMA protein expression (brown) in lung tissue sections. Representative images from each group and AOD value are shown (B). The data are shown as the mean ± SD (n =3). *P<0.05, **P<0.01, ***P<0.001, ****P<0.0001.

MEL: Melittin; BLM: Bleomycin; EMT: Epithelial-mesenchymal transition; WB: Western blot; AOD: Average optical density ECM: Extracellular matrix

Serum T-AOC activity (A), GSH-PX activity (B), and MDA content (C) were measured with commercial kits. Mitochondrial function was assessed by measuring mitochondrial respiratory chain complex I-V activity (D-H) and the ATP level (I). The data are shown as the mean ± SD (n =6). *P<0.05, **P<0.01, ***P<0.001, ****P<0.0001.

MEL: Melittin; BLM: Bleomycin; OS: Oxidative stress; T-AOC: Total anti-oxidant capacity; GSH-PX: Glutathione peroxidase

Images of immunohistochemical staining for TGF-β1 and evaluation of positive TGF-β1 expression (A) in tissues. The levels of proteins associated with the TGF-β1/Smad2/3 (B-E) and AMPK/SIRT1/PGC-1α (F-I) signaling pathways were measured by WB. The data are shown as the mean ± SD (n =3). *P<0.05, **P<0.01, ***P<0.001, ****P<0.0001.

MEL: Melittin; PF: Pulmonary fibrosis

## Conclusion

In summary, MEL is protective against BLM-induced PF. MEL may not only inhibit EMT and ECM deposition by regulating the TGF-β1/Smad2/3 pathway but also improve mitochondrial energy metabolism, maintain mitochondrial function, and reduce OS by activating the AMPK/SIRT1/PGC-1α pathway.

## Declaration

We have not used any AI tools or technologies to prepare this manuscript.

## References

[1] Podolanczuk AJ, Thomson CC, Remy-Jardin M, Richeldi L, Martinez FJ, Kolb M (2023). Idiopathic pulmonary fibrosis: state of the art for 2023. Eur Respir J.

[2] Andugulapati SB, Gourishetti K, Tirunavalli SK, Shaikh TB, Sistla R (2020). Biochanin-A ameliorates pulmonary fibrosis by suppressing the TGF-β mediated EMT, myofibroblasts differentiation and collagen deposition in in vitro and in vivo systems. Phytomedicine.

[3] Li Y, Qin W, Liang Q, Zeng J, Yang Q, Chen Y (2023). Bufei huoxue capsule alleviates bleomycin-induced pulmonary fibrosis in mice via TGF-β1/Smad2/3 signaling. J Ethnopharmacol.

[4] Ji Y, Dou YN, Zhao QW, Zhang JZ, Yang Y, Wang T (2016). Paeoniflorin suppresses TGF-β mediated epithelial-mesenchymal transition in pulmonary fibrosis through a Smad-dependent pathway. Acta Pharmacol Sin.

[5] Otoupalova E, Smith S, Cheng G, Thannickal VJ (2020). Oxidative Stress in Pulmonary Fibrosis. Compr Physiol.

[6] Mori MP, Penjweini R, Knutson JR, Wang PY, Hwang PM (2022). Mitochondria and oxygen homeostasis. FEBS J.

[7] Gazdhar A, Lebrecht D, Roth M, Tamm M, Venhoff N, Foocharoen C (2014). Time-dependent and somatically acquired mitochondrial DNA mutagenesis and respiratory chain dysfunction in a scleroderma model of lung fibrosis. Sci Rep.

[8] Yu G, Tzouvelekis A, Wang R, Herazo-Maya JD, Ibarra GH, Srivastava A (2018). Thyroid hormone inhibits lung fibrosis in mice by improving epithelial mitochondrial function. Nat Med.

[9] Zhang N, Li P, Lin H, Shuo T, Ping F, Su L (2021). IL-10 ameliorates PM2 5-induced lung injury by activating the AMPK/SIRT1/PGC-1α pathway. Environ Toxicol Pharmacol.

[10] Mansour HH, Omran MM, Hasan HF, El Kiki SM (2020). Modulation of bleomycin-induced oxidative stress and pulmonary fibrosis by N-acetylcysteine in rats via AMPK/SIRT1/NF-κβ. Clin Exp Pharmacol Physiol.

[11] Cantó C, Gerhart-Hines Z, Feige JN, Lagouge M, Noriega L, Milne JC (2009). AMPK regulates energy expenditure by modulating NAD+ metabolism and SIRT1 activity. Nature.

[12] Thirupathi A, de Souza CT (2017). Multi-regulatory network of ROS: the interconnection of ROS, PGC-1 alpha, and AMPK-SIRT1 during exercise. J Physiol Biochem.

[13] Guha S, Ferrie RP, Ghimire J, Ventura CR, Wu E, Sun L (2021). Applications and evolution of melittin, the quintessential membrane active peptide. Biochem Pharmacol.

[14] Shi P, Xie S, Yang J, Zhang Y, Han S, Su S (2022). Pharmacological effects and mechanisms of bee venom and its main components: Recent progress and perspective. Front Pharmacol.

[15] El-Aarag B, Magdy M, AlAjmi MF, Khalifa SAM, El-Seedi HR (2019). Melittin exerts beneficial effects on paraquat-induced lung injuries in mice by modifying oxidative stress and apoptosis. Molecules.

[16] Li L, Zhang S, Wei L, Wang Z, Ma W, Liu F (2020). Antifibrotic effect of melittin on TRIM47 expression in human embryonic lung fibroblast through regulating TRIM47 pathway. Life Sci.

[17] Park JH, Park B, Park KK (2017). Suppression of hepatic epithelial-to-mesenchymal transition by melittin via blocking of TGFβ/Smad and MAPK-JNK signaling pathways. Toxins (Basel).

[18] Szapiel SV, Elson NA, Fulmer JD, Hunninghake GW, Crystal RG (1979). Bleomycin-induced interstitial pulmonary disease in the nude, athymic mouse. Am Rev Respir Dis.

[19] Savin IA, Zenkova MA, Sen’kova AV (2022). Pulmonary fibrosis as a result of acute lung inflammation: molecular mechanisms, relevant in vivo models, prognostic and therapeutic approaches. Int J Mol Sci.

[20] Ju N, Hayashi H, Shimamura M, Baba S, Yoshida S, Morishita R (2022). Prevention of bleomycin-induced pulmonary fibrosis by a RANKL peptide in mice. Sci Rep.

[21] Ding Y, Wang L, Liu B, Ren G, Okubo R, Yu J (2022). Bryodulcosigenin attenuates bleomycin-induced pulmonary fibrosis via inhibiting AMPK-mediated mesenchymal epithelial transition and oxidative stress. Phytother Res.

[22] Li S, Yang Q, Chen F, Tian L, Huo J, Meng Y (2022). The antifibrotic effect of pheretima protein is mediated by the TGF-β1/Smad2/3 pathway and attenuates inflammation in bleomycin-induced idiopathic pulmonary fibrosis. J Ethnopharmacol.

[23] Wang XC, Song K, Tu B, Sun H, Zhou Y, Xu SS (2023). New aspects of the epigenetic regulation of EMT related to pulmonary fibrosis. Eur J Pharmacol.

[24] Salton F, Volpe MC, Confalonieri M (2019). Epithelial-mesenchymal transition in the pathogenesis of idiopathic pulmonary fibrosis. Medicina (Kaunas).

[25] Hu HH, Chen DQ, Wang YN, Feng YL, Cao G, Vaziri ND (2018). New insights into TGF-β/Smad signaling in tissue fibrosis. Chem Biol Interact.

[26] Lv Q, Wang J, Xu C, Huang X, Ruan Z, Dai Y (2020). Pirfenidone alleviates pulmonary fibrosis in vitro and in vivo through regulating Wnt/GSK-3β/β-catenin and TGF-β1/Smad2/3 signaling pathways. Mol Med.

[27] Qian W, Cai X, Qian Q, Zhang W, Wang D (2018). Astragaloside IV modulates TGF-β1-dependent epithelial-mesenchymal transition in bleomycin-induced pulmonary fibrosis. J Cell Mol Med.

[28] Ma WH, Li M, Ma HF, Li W, Liu L, Yin Y (2020). Protective effects of GHK-Cu in bleomycin-induced pulmonary fibrosis via anti-oxidative stress and anti-inflammation pathways. Life Sci.

[29] An HJ, Kim JY, Kim WH, Han SM, Park KK (2016). The protective effect of melittin on renal fibrosis in an animal model of unilateral ureteral obstruction. Molecules.

[30] Fois AG, Paliogiannis P, Sotgia S, Mangoni AA, Zinellu E, Pirina P (2018). Evaluation of oxidative stress biomarkers in idiopathic pulmonary fibrosis and therapeutic applications: a systematic review. Respir Res.

[31] Pan L, Cheng Y, Yang W, Wu X, Zhu H, Hu M (2023). Nintedanib ameliorates bleomycin-induced pulmonary fibrosis, inflammation, apoptosis, and oxidative stress by modulating PI3K/Akt/mTOR pathway in mice. Inflammation.

[32] Balaha M, Alahmari A, Kandeel S, Balaha M (2023). Vinpocetine’s immunomodulating, antioxidant, anti-inflammatory, ant-ifibrotic, and PDE inhibiting potencies ameliorate bleomycin-induced pulmonary fibrosis. Iran J Basic Med Sci.

[33] Yaghoubi A, Amel Jamehdar S, Reza Akbari Eidgahi M, Ghazvini K (2022). Evaluation of the therapeutic effect of melittin peptide on the ulcerative colitis mouse model. Int Immunopharmacol.

[34] Nguyen CD, Lee G (2021). Neuroprotective activity of melittin-the main component of bee venom-against oxidative stress induced by Aβ25-35 in in vitro and in vivo models. Antioxidants (Basel).

[35] Siekacz K, Piotrowski WJ, Iwański MA, Górski P, Białas AJ (2021). The role of interaction between mitochondria and the extracellular matrix in the development of idiopathic pulmonary fibrosis. Oxid Med Cell Longev.

[36] Larson-Casey JL, He C, Carter AB (2020). Mitochondrial quality control in pulmonary fibrosis. Redox Biol.

[37] Guan S, Zhao L, Peng R (2022). Mitochondrial respiratory chain supercomplexes: from structure to function. Int J Mol Sci.

[38] Zank DC, Bueno M, Mora AL, Rojas M (2018). Idiopathic pulmonary fibrosis: Aging, mitochondrial dysfunction, and cellular bioenergetics. Front Med (Lausanne).

[39] Rabinovitch RC, Samborska B, Faubert B, Ma EH, Gravel SP, Andrzejewski S (2017). AMPK maintains cellular metabolic homeostasis through regulation of mitochondrial reactive oxygen species. Cell Rep.

[40] Yang X, Liu Q, Li Y, Tang Q, Wu T, Chen L (2020). The diabetes medication canagliflozin promotes mitochondrial remodelling of adipocyte via the AMPK-Sirt1-Pgc-1α signalling pathway. Adipocyte.

[41] Tian L, Cao W, Yue R, Yuan Y, Guo X, Qin D (2019). Pretreatment with Tilianin improves mitochondrial energy metabolism and oxidative stress in rats with myocardial ischemia/reperfusion injury via AMPK/SIRT1/PGC-1 alpha signaling pathway. J Pharmacol Sci.

[42] Rangarajan S, Bone NB, Zmijewska AA, Jiang S, Park DW, Bernard K (2018). Metformin reverses established lung fibrosis in a bleomycin model. Nat Med.

[43] Chien LH, Deng JS, Jiang WP, Chou YN, Lin JG, Huang GJ (2023). Evaluation of lung protection of Sanghuangporus sanghuang through TLR4/NF-κB/MAPK, keap1/Nrf2/HO-1, CaMKK/AMPK/Sirt1, and TGF-β/SMAD3 signaling pathways mediating apoptosis and autophagy. Biomed Pharmacother.

[44] Li Z, Jiao Y, Wu Z, Liu H, Li Y, Cai Y (2024). The role of quercetin in ameliorating bleomycin-induced pulmonary fibrosis: insights into autophagy and the SIRT1/AMPK signaling pathway. Mol Biol Rep.

